# Transformation of Inferior Tomato into Preservative: Fermentation by Multi-Bacteriocin Producing *Lactobacillus paracasei* WX322

**DOI:** 10.3390/foods10061278

**Published:** 2021-06-03

**Authors:** Rong Zhu, Xiaoqing Liu, Xiaofen Li, Kaifang Zeng, Lanhua Yi

**Affiliations:** 1 College of Food Science, Southwest University, Chongqing 400715, China; zr852963@email.swu.edu.cn (R.Z.); lyj594lxq@email.swu.edu.cn (X.L.); lixiaofen222@email.swu.edu.cn (X.L.); zengkaifang@hotmail.com (K.Z.); 2 Research Center of Food Storage & Logistics, Southwest University, Chongqing 400715, China

**Keywords:** tomato, bacteriocin, soft rot, biocontrol, vegetable preservation

## Abstract

Loss and waste of postharvest vegetables are the main challenges facing the world’s vegetable supply. In this study, an innovative method of value-added transformation was provided: production of bacteriocin from vegetable waste, and then its application to preservation of vegetables. Antibacterial activity to soft rot pathogen *Pectobacterium cartovorum* (*Pcb* BZA12) indicated that tomato performed best in the nutrition supply for bacteriocin production among 12 tested vegetables. Moreover, the antibacterial activity was from *Lactobacillus paracasei* WX322, not components of vegetables. During a fermentation period of 10 days in tomato juice, *L. paracasei* WX322 grew well and antibacterial activity reached the maximum on the tenth day. Thermostability and proteinase sensitivity of the bacteriocin from tomato juice were the same with that from Man-Rogosa-Sharpe broth. Scanning electron microscope images indicated that the bacteriocin from tomato juice caused great damage to *Pcb* BZA12. At the same time, the bacteriocin from tomato juice significantly reduced the rotten rate of Chinese cabbage from 100% ± 0% to 20% ± 8.16% on the third day during storage. The rotten rate decrease of cucumber, tomato, and green bean was 100% ± 0% to 0% ± 0%, 70% ± 14.14% to 13.33% ± 9.43%, and 76.67% ± 4.71% to 26.67% ± 4.71%, respectively. Bacteriocin treatment did not reduce the rotten rate of balsam pear, but alleviated its symptoms.

## 1. Introduction

Vegetables, as an essential food in people’s daily life, can provide dietary fiber, minerals, vitamins, and other nutrients for the human body [1]. The health benefits of vegetable consumption include promoting gastrointestinal peristalsis [2], preventing chronic diseases (such as high blood pressure, diabetes, dementia) [3], boosting immunity [4], and so on. Experts of the United Nations Food and Agriculture Organization (FAO) and the World Health Organization (WHO) have encouraged people to eat more vegetables and fruits to reduce the risk of chronic diseases and cancer [5]. Global vegetable production is increasing, but it is still hard to meet the recommended intake (≥240 g/day according to WHO), especially in developing countries. For example, mean vegetable intake is 71 g/day in Melanesia [6], far less than the recommendation. According to the reports of FAO, a real challenge laid before the world’s vegetable supply is postharvest loss and waste. Fruit and vegetable waste is one of the main food wastes, representing 0.5 billion tons per year (FAO 2011). In the US, 9% of fruit and 8% of vegetables are lost at the retail stage, and a further 19% of fruit and 22% of vegetables are not eaten at the consumption stage [7]. These wastes are not only critical at the agricultural stage in developing countries, but they are also high at the processing stage [8,9]. Waste from harvest inferior products and processing by-products accounts for about 30% of whole vegetables [10]. Therefore, innovative approaches and technology in combating postharvest loss and waste of vegetables are urgently needed.

Globally, postharvest loss of vegetables is as high as 60% (usually 20–60%) of total vegetable production [7]. Postharvest losses of vegetables are mainly caused by physiological disorders and infectious diseases. Infectious diseases of postharvest vegetables include bacterial diseases and fungal diseases, the former has caused more losses in leafy vegetables. Bacterial soft rot is a destructive disease, which causes more serious losses than any other bacterial disease [11]. For example, approximately 22% of potatoes are lost per year, among which bacterial soft rot alone accounts for 30–50% [12]. Bacterial pathogens leading to soft rot of vegetables include *Pectobacterium* [12], *Dickeya* [13], *Pseudomonas* [14], *Xanthomonas* [15], etc. *Pectobacterium carotovorum*, formerly *Erwinia carotovorum*, is the most commonly reported pathogen of vegetable soft rot and lists among the top 10 plant pathogenic bacteria [16]. Soft rot caused by *P. carotovorum* can occur anytime during postharvest handling, especially storage and transportation. *P. carotovorum* has a very broad range of hosts, including Solanaceae, Cruciferae, Cucurbitaceae, Compositae, Liliaceae, etc., involving almost all kinds of vegetables. The huge economic loss derived from soft rot has triggered great effort of researchers to find methods to control *P. carotovorum* in vegetables.

A lot of vegetable waste is generated during the processes of agricultural production, postharvest handling and storage, including inferior products and/or by-products, especially for the processing of fresh-cut vegetables [17]. Strategies of vegetable waste management have been reviewed by Plazzotta et al. [18], Esparza et al. [19], among others. Conventional waste management technologies consist of animal feeding, soil amendment, composting, and anaerobic digestion (biogas). Value-added applications contain extraction of bioactive compounds (e.g., flavonoids, phenolic acids, terpenes, oils, dietary fiber), production of enzymes, production of exopolysaccharides, production of biofuels, synthesis of bioplastics. Like the production of enzymes, vegetable wastes also can be used to produce antimicrobials through providing nutrition for the growth of producer microorganisms, which can be applied to control soft rot caused by *P. carotovorum*. However, few works have focused on such a transformation.

Bacteriocin is a kind of antibacterial polypeptide or precursor polypeptide synthesized by ribosomes in the process of bacteria metabolism [20]. It has ideal antibacterial properties and inhibits the growth of some food pathogenic and spoilage bacteria without side effects as a food preservative [21,22]. Nevertheless, bacteriocin is rarely reported to control soft rot of postharvest vegetables. In our previous study, a multi-bacteriocin producing lactic acid bacteria, *Lactobacillus paracasei* WX322, was isolated from fermented vegetable (pickle) [23] with good antibacterial activity against *P. carotovorum*. Bacteriocins from lactic acid bacteria are usually produced in Man-Rogosa-Sharpe broth with high cost. Pickle inspires us that vegetable wastes can be used to provide nutrition for the growth and bacteriocin production of *L. paracasei* WX322. Previous results indicated that fermentation products of *L. paracasei* WX322 in some vegetables could inhibit the growth of *P. carotovorum* (not published). The aims of this study were: to find appropriate vegetable waste to produce bacteriocins with higher activity than Man-Rogosa-Sharpe broth (to alternative to Man-Rogosa-Sharpe broth), then to analyze the growth property and bacteriocin production of *L. paracasei* WX322 in tomato juice, and finally to investigate the control potential of bacteriocin products from tomato juice fermentation to soft rot of different vegetables. This study can not only provide a new way to transform inferior tomato into value-added product (food preservative), but also provide a biocontrol approach with high safety to reduce postharvest losses of vegetables.

## 2. Materials and Methods

### 2.1. Antibacterial Activity of L. paracasei WX322 Fermentation Products from 12 Kinds of Vegetables

#### 2.1.1. Screening of Vegetable Fermentation with Good Antibacterial Activity

*L. paracasei* WX322 (CGMCC No.17710) was preserved in our lab. *Pectobacterium cartovorum* subsp. *brasilliense* BZA12 (*Pcb* BZA12) was donated by Prof. Changyuan Liu from Liaoning Academy of Agricultural Sciences. Vegetables of tomato (*Solanum lycopersicum*), cucumber (*Cucumis sativus*), Chinese cabbage (*Brassica rapa* ssp. pekinensis), soybean sprouts (*Glycine max* L. Merrill), mungbean sprout (*Vigna radiate* L. Wilzcek), okra (*Abelmoschus esculentus* L. Moench), garlic chives (*Allium tuberosum* Rottl. ex Spreng), green bean (*Phaseolus vulgaris*), carrot (*Daucus carota*), balsam pear (*Momordica charantia*), white ground (*Benincasa hispida*), purple cabbage (*Brassica oleracea* L. var. Capitata) were collected from the local supermarket of Beibei, Chongqing, China.

In order to find a viable alternative to Man-Rogosa-Sharpe broth to produce bacteriocin, inferior products losing the commodity value of the 12 vegetables above were squeezed into juice. Specifically, vegetables were washed using running water, and then cut into small pieces using a knife. The pretreated vegetables were put into a Juicer WBL2501B (Midea, Guangdong, China) and squeezed with appropriate water. Juices of different vegetables were sterilized at 121 °C for 15 min. *L. paracasei* WX322 was inoculated into 100 mL sterile Man-Rogosa-Sharpe broth and cultured at 37 °C under 120 rpm for 24 h to logarithmic phase as seed. The seed of *L. paracasei* WX322 was inoculated into each prepared vegetable juice (100 mL) at a dose of 1%, and then stationarily cultured at 30 °C for 10 days. Subsequently, the fermentation product of each vegetable juice was centrifuged (5000 rpm, 15 min, 25 °C) using a high-speed freezing centrifuge ALLEGRA X-15R (Beckman Coulter Inc., CA, USA). The supernatant was filtered (0.22 μM pore size) and concentrated five folds in a vacuum freeze dryer LGJ-10 (Beijing Songyuan Huaxing Technology Develop Co., Ltd., Beijing, China). The antibacterial activity of the concentrated samples against *Pcb* BZA12 was determined by the agar well diffusion method as in our previous study [24]. The same treatment with Man-Rogosa-Sharpe broth was used as the control. All treatments were carried out for three times.

#### 2.1.2. Verification of Antibacterial Activity from *L. paracasei* WX322 Metabolites

Components of some fruits and vegetables have been reported to possess antibacterial activity [25,26]. To eliminate the possibility that the antibacterial activity was from vegetable components, vegetable juice fermentation with and without *L. paracasei* WX322 were simultaneously undertaken. Tomato, Chinese cabbage, mungbean sprout, purple cabbage and okra were tested, and the fermentation process was carried out as described above. Supernatant without concentration was applied to test antibacterial activity, in which *Pcb* BZA12 was used as the indicator.

### 2.2. Microbial Growth of L. paracasei WX322 in Tomato Juice and Antibacterial Activity Analysis

The seed of *L. paracasei* WX322 was inoculated into prepared tomato juice at a ratio of 1%, and then incubated at 30 °C in stationary status. Sample was taken out each day, and then applied to analysis of microbial growth and antibacterial activity. Three replications of the tomato juice fermentation were conducted.

For microbial growth measurement of *L. paracasei* WX322 in tomato juice, the juice was mixed well and a 1 mL sample was taken out. The sample was diluted 10-fold using sterile saline (0.9% NaCl) from 10^−1^ to 10^−8^. Each dilution was spread on Man-Rogosa-Sharpe agar plate. The plates were incubated at 37 °C for 48 h, and then colony number was counted.

For antibacterial activity measurement of fermentation supernatant, the juice was mixed well and 1 mL sample was taken out. The supernatant was obtained by centrifugation and its antibacterial activity was determined by the agar well diffusion method, in which *Pcb* BZA12 was used as the indicator.

### 2.3. Sensitivity of Fermentation Product of L. paracasei WX322 in Tomato Juice to Heat and Proteinases

*L. paracasei* WX322 was inoculated in sterile tomato juice and incubated at 30 °C for 10 days. The supernatant was obtained by centrifugation and subjected to assays of sensitivity analysis to heat and proteinases referring to Qi et al. [11].

For sensitivity analysis of fermentation product to heat, the sample was heated at 60 °C, 80 °C, 100 °C, 120 °C for 10 min, 20 min, 30 min, respectively. Among temperatures, 60 °C and 80 °C were conducted in a Digital Thermostat Water Bath HH-6 (LiChen, Shanghai Lichen Bangxi Instrument Technology Co., Ltd., Shanghai, China), 100 °C and 120 °C in a vertical autoclave G154D (Zealway, Xiamen Zealway Instrument Inc., Xiamen, China). After treatment, the samples were cooled to room temperature (about 20 °C), and the residual antibacterial activity was measured using the agar well diffusion method. The indicator used was *Pcb* BZA12. Unheated sample was used as the control.

For sensitivity analysis of the fermentation product to proteases, pepsin and proteinase K were used. Pepsin and proteinase K were dissolved in corresponding optimal buffers at 2 mg/mL: pH 3.0 citric acid-sodium citrate buffer, pH 7.0 Tris-HCl buffer, respectively. Then, the enzyme solution was added into samples at a final concentration of 1 mg/mL. After incubated at 37 °C (pepsin) or 55 °C (proteinase K) for 4 h, the residual antibacterial activity was determined using the agar well diffusion method. The indicator used was *Pcb* BZA12. Fermentation product without enzyme treatment and enzyme buffers without fermentation product were used as controls.

### 2.4. Preparation of Bacteriocin Sample and Its Antibacterial Activity Determination

*L. paracasei* WX322 was cultured in 3 L tomato juice at 30 °C for 10 days to prepare the bacteriocin sample. Subsequently, soluble polysaccharide from tomato was removed by alcohol precipitation [27,28]. Specifically, supernatant was obtained by centrifugation and ethanol was gradually added into the supernatant up to 80%. After stirred overnight, the precipitated saccharides were removed by centrifugation and ethanol in the supernatant was removed by rotary evaporation on a Rotary Evaporator RE-52AA (YaRong, Shanghai Yarong Biochemical instrument Factory, Shanghai, China) at 45 °C and 0.07 MPa. Moreover, microporous adsorption resin D101 was used to get rid of pigment in the sample. Antibacterial activity of the bacteriocin sample was measured using the agar well diffusion method after serial 2-fold dilution and expressed in arbitrary units (AU) per milliliter as described by Sabo et al. [29].

### 2.5. Scanning Electron Microscope (SEM) Observation

To investigate the effect of bacteriocin sample on cell morphology of *Pcb* BZA12, SEM was used referring to Li et al. [30]. Cells of *Pcb* BZA12 were collected in mid-logarithmic phase (OD_600nm_ ≈ 0.2) by centrifugation at 5000 rpm for 15 min. Then, cells were resuspended in fresh LB broth and bacteriocin sample was added with a final concentration of 320 AU/mL. After treatment for 2 h at 37 °C, cells were washed with sterile saline three times to remove the residual bacteriocin sample. Subsequently, cells were fixed on glass slides using 2.5% (*v*/*v*) glutaraldehyde at 4 °C for 12 h. Further, cells were washed with sterile water and gradually dehydrated in ethanol solutions of 50%, 70%, 80%, 90%, 100%, and then in 100% acetone. Finally, the sample on the glass slide was dried completely and the morphology change of cells after bacteriocin sample treatment was observed by a Phenom Pro10102 scanning electron microscopy (Phenom-World, Eindhoven, The Netherlands). Areas with homogeneous cells were selected to take pictures at 10,000× *g* magnification. The same treatment with sterile saline instead of bacteriocin sample was used as the control.

### 2.6. Controlling Bacterial Soft Rot of Five Vegetables

Vegetables of Chinese cabbage, green bean, tomatoes, cucumber and balsam pear were purchased from local supermarket. Vegetables were treated as follows: Chinese cabbage and green bean were drilled on the surface with a sterile punch, tomatoes surface was peeled off in a small area, and cucumber and balsam pear were chopped to 5 cm in length. Bacterial cells of *Pcb* BZA12 were collected in mid-logarithmic phase and resuspended in sterile saline to OD_600nm_ = 0.2 using an Ultraviolet-visible Spectrophotometer V-5000 (METASH, Shanghai Yuanxi Instrument Co., Ltd., Shanghai, China). Then, 20 μL cell suspension of *Pcb* BZA12 was added into each well or onto the cut area. Vegetables were allowed to air dry at room temperature for 3 h. The vegetables inoculated with *Pcb* BZA12 were divided into two groups. For one group, 20 μL of 320 AU/mL bacteriocin sample was added into each well or onto the cut area. For the other group, 20 μL sterile water instead of bacteriocin sample was added as the control. Each group of each vegetable had 10 produces, which was conducted with 3 replications. All treated vegetables were allowed to air dry at room temperature for 3 h. Finally, vegetables were packaged in bags and placed at 30 °C to observe the incidence of soft rot.

### 2.7. Statistical Analysis

Three biological replicates were performed for all experiments and the results were expressed as mean ± standard deviation. Figures were carried out with OriginPro 8.1 (OriginLab, Northampton, MA, USA). Experimental differences were determined using a one-way analysis of variance (ANOVA) and Duncan test at 0.05 levels.

## 3. Results and Discussion

### 3.1. Screening Vegetable Alternative to Man-Rogosa-Sharpe Broth among 12 Kinds of Vegetables

*P. carotovorum* is generally known as a tissue maceration agent and causes bacterial soft rot disease [31], which has led to huge economic losses of postharvest vegetables. With the improvement of awareness of environmental protection and health, biological methods are more preferred by consumers to control soft rot of postharvest vegetables. Lactic acid bacteria are generally recognized as safe (GRAS) and have been used to combat with diseases of fruits and vegetables in both preharvest and postharvest in recent years. For example, *Lactobacillus plantarum* strain BY was used to control the soft rot of Chinese cabbage under field conditions [32] and *Lactobacillus sucicola* JT03 was used to control green mold of postharvest citrus [33]. Our previous study indicated that *L. paracasei* WX322 had good inhibitory activity against *P. carotovorum* in vitro and the bacteriocin from *L. paracasei* WX322 played a key role [11]. However, Man-Rogosa-Sharpe broth is not appropriate to be used directly in vegetables because it will affect the flavor and color of vegetables. Moreover, the cost of Man-Rogosa-Sharpe broth is high. Therefore, we aimed to seek low-cost and healthy nutrition provider for the production of bacteriocin by *L. paracasei* WX322.

Antibacterial activity of fermented products by *L. paracasei* WX322 from different vegetable juices after 5-fold concentration is shown in Table 1. Man-Rogosa-Sharpe broth was used as the reference whose inhibition zone diameter to *P. carotovorum* was 40.5 ± 0.4 mm. It is interesting that the fermentation products of two kinds of vegetables, namely tomato and cucumber, had higher inhibitory activity than Man-Rogosa-Sharpe broth, especially tomato with an inhibition zone diameter 47.3 ± 1.6 mm. The other 10 kinds of vegetables also had good antibacterial activity with inhibition zone diameter ranging from 37.4 ± 1.2 mm to 40.3 ± 0.4 mm. Lactic acid bacteria are usually a small part of the autochthonous microbiota of vegetables and fruits [34]. *L. paracasei* WX322 was isolated from homemade pickle [23], which is also an autochthonous lactic acid bacteria of vegetables. As we expected, vegetables can be used to provide nutrition for the bacteriocin production of *L. paracasei* WX322. Among 12 kinds of vegetables tested, tomato was the best nutrition provider. Tomato is grown and consumed all over the world with yield over 180 million tonnes per year according to the FAO. The generation of inferior products and by-products is inevitable during tomato planting and processing. However, more works are focused on value-added utilization of tomato pomace [35]. Attention also needs to be paid to the application of inferior tomato. In this study, production of bacteriocin by tomato juice fermentation from inferior products also provides another value-added utilization of tomato waste.

**Figure 1:**
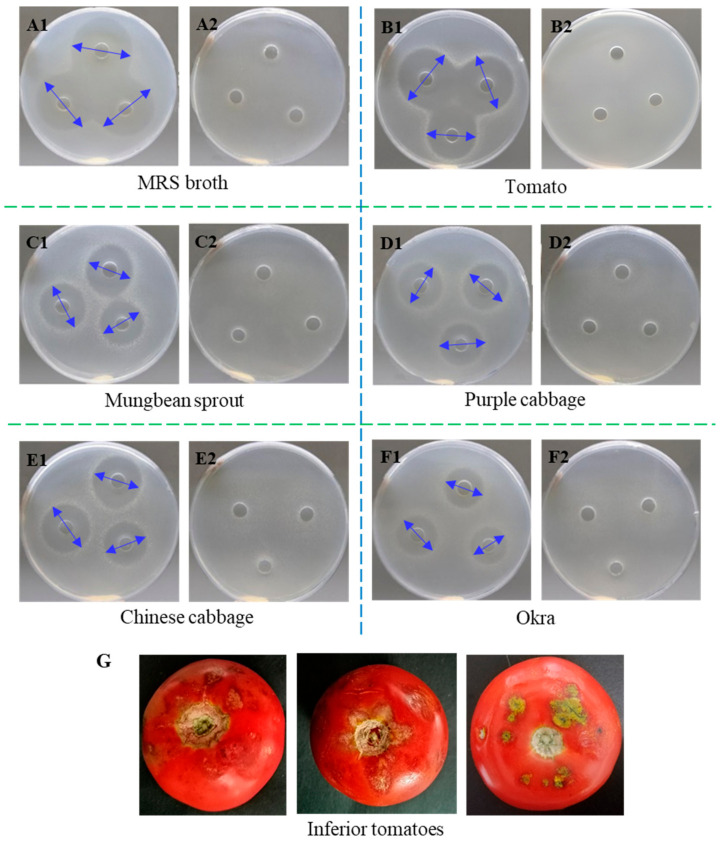
Antibacterial activity of fermentation supernatant. (**A1**), Man-Rogosa-Sharpe broth with *L. paracasei* WX322 as a reference; (**A2**), Man-Rogosa-Sharpe broth without *L. paracasei* WX322 as a reference. (**B1**), Tomato with *L. paracasei* WX322; (**B2**), tomato without *L. paracasei* WX322. (**C1**), Mungbean sprout with *L. paracasei* WX322; (**C2**), mungbean sprout without *L. paracasei* WX322. (**D1**), Purple cabbage with *L. paracasei* WX322; (**D2**), purple cabbage without *L. paracasei* WX322. (**E1**), Chinese cabbage with *L. paracasei* WX322; (**E2**), Chinese cabbage without *L. paracasei* WX322. (**F1**), Okra with *L. paracasei* WX322; (**F2**), okra without *L. paracasei* WX322. (**G**), Inferior products of tomato as an example. Inhibition zone diameter was marked by blue arrows.

Components of some fruits and vegetables also have antimicrobial activity [25,26]. Although great antibacterial activity of fermented products was observed in Table 1, it is unclear that whether the inhibition action was from metabolites produced by *L. paracasei* WX322 or from both components of vegetables and lactic acid bacteria metabolites. Therefore, antibacterial activity of five kinds of vegetable fermentations without *L. paracasei* WX322 was analyzed. As shown in Figure 1, the reference (A2, Man-Rogosa-Sharpe broth) and vegetables (B2, C2, D2, E2, F2) without *L. paracasei* WX322 had no inhibitory activity against *P. carotovorum*. In contrast, all fermentations with *L. paracasei* WX322 (A1, B1, C1, D1, E1, F1 in Figure 1) exhibited obvious inhibition zone. The result indicated that antibacterial activity of fermentation supernatant came from metabolites of *L. paracasei* WX322 not components of vegetables. Namely, vegetable juice only provided nutrition for the growth and bacteriocin production of *L. paracasei* WX322. Production of active peptides by tomato fermentation using microorganism has also been reported by other studies. For example, tomato waste was reported to produce antioxidant and ACE-inhibitory peptides by fermentation using *Bacillus subtilis* [36]. Therefore, the juice from inferior tomato (Figure 1G) was selected as medium for bacteriocin production by *L. paracasei* WX322 to alternative to Man-Rogosa-Sharpe broth.

### 3.2. Microbial Growth of L. paracasei WX322 and Antibacterial Activity of Fermented Product in Tomato Juice

**Figure 2:**
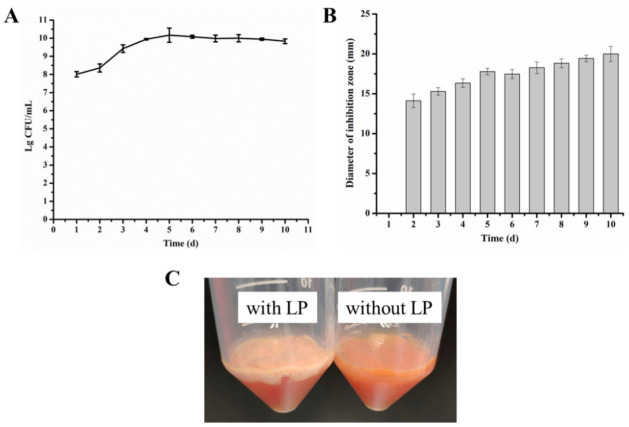
Fermentation characteristics of tomato juice by *L. paracasei* WX322. (**A**), Microbial number of *L. paracasei* WX322; (**B**), antibacterial activity of fermentation supernatant; (**C**), residues of fermented tomato juice after centrifugation, LP is *L. paracasei* WX322.

In order to understand fermentation characteristics of *L. paracasei* WX322 in tomato juice, a dynamic process of microbial growth was investigated and the antibacterial activity of the fermentation product was measured. As shown in Figure 2A, the number of *L. paracasei* WX322 increased in the first 5 days and reached a maximum of about 10.19 log_10_ colony-forming units (cfu)/mL on the fifth day. Subsequently, a slight decrease of microbial number was observed, which may derive from the gradual consumption of nutrients and accumulation of metabolites. At the same time, tomato juice on the bottom was collected and its residues after centrifugation was observed. According to Figure 2C, residues of fermented tomato juice with *L. paracasei* WX322 was covered by white cells (left tube) while that without *L. paracasei* WX322 had no white (right tube). This result also revealed the great growth of *L. paracasei* WX322 in tomato juice.

Fermentation product of *L. paracasei* WX322 using tomato juice exhibited antibacterial activity on the second day (Figure 2B), which was close to our previous study using Man-Rogosa-Sharpe broth in which bacteriocin was produced after fermentation for 36 h [11]. Moreover, with the extension of fermentation time, antibacterial activity of fermentation supernatant gradually increased. On the tenth day, the inhibition zone diameter of fermented tomato juice supernatant reached the maximum. Bacteriocins are usually secondary metabolites [37] and accumulate extracellularly with the cell density increase because of quorum sensing [38,39]. During the fermentation period of 10 days, the fermented product on the last day had the highest activity, therefore, 10-day was used as the fermentation time for tomato juice to produce bacteriocin by *L. paracasei* WX322.

### 3.3. Sensitivity of Fermentation Product of L. paracasei WX322 from Tomato Juice to Heat and Proteinase

**Figure 3:**
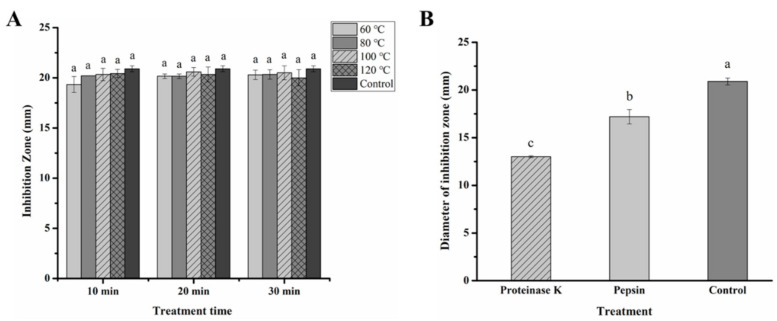
Sensitivity of fermentation product of *L. paracasei* WX322 from tomato juice to heat (**A**) and proteinase (**B**). (**A**), Fermentation product was heated at 60 °C, 80 °C, 100 °C, 120 °C for 10 min, 20 min, 30 min, respectively. (**B**), Fermentation product was treated by proteinases. Letters of a, b, c are significantly different by analysis of variance (ANOVA) and Tukey’s test (*p* < 0.05).

To make a further comparison of the bacteriocin produced by *L. paracasei* WX322 from Man-Rogosa-Sharpe broth and tomato juice, its sensitivity to heat and proteinases was analyzed. As shown in Figure 3A, the fermentation product from tomato juice had excellent thermostability that no significant difference was found in different time and temperature treatments. It still had good antibacterial activity even treated at 120 °C for 30 min. The excellent thermostability of bacteriocin of *L. paracasei* WX322 from tomato juice in this study remained the same as it was from Man-Rogosa-Sharpe broth in our previous study [11].

At the same time, sensitivity of fermentation product from tomato juice to proteinase K and pepsin in this study was also in line with that from Man-Rogosa-Sharpe broth in our previous study [11]. Namely, treatments by proteinase K and pepsin caused partial loss of antibacterial activity.

In short, characteristics of the bacteriocin produced by *L. paracasei* WX322 from tomato juice are identical to those from Man-Rogosa-Sharpe broth. Therefore, tomato juice can be used to replace Man-Rogosa-Sharpe broth to produce bacteriocin by *L. paracasei* WX322.

### 3.4. Effect of Bacteriocin of L. paracasei WX322 from Tomato Juice on Cell Morphology of Pcb BZA12

A bacteriocin sample produced by *L. paracasei* WX322 was prepared by fermentation in tomato juice. According to the report from Williams and Bevenue [28], tomato solids insoluble in 80% ethyl alcohol are primarily polysaccharides. Therefore, 80% ethanol was used to remove polysaccharides from tomato. At the same time, some undesired pigments were removed by resin. Finally, antibacterial activity of the obtained bacteriocin sample was 640 AU/mL.

**Figure 4:**
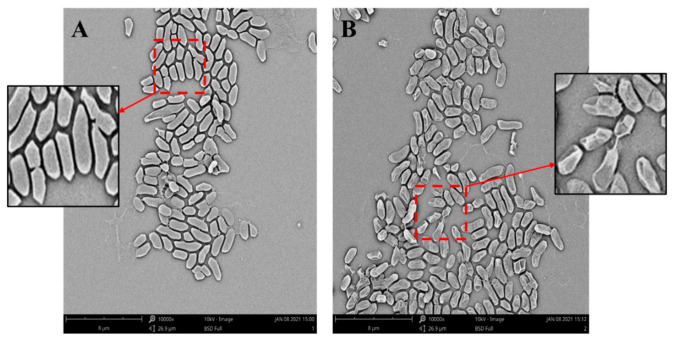
Scanning electron microscope (SEM) images of *Pcb* BZA12. (**A**), control with sterile saline; (**B**), treatment with bacteriocin of *L. paracasei* WX322 from tomato juice.

To observe the effect of the bacteriocin produced by *L. paracasei* WX322 from tomato juice on cell morphology of *Pcb* BZA12, 320 AU/mL sample was used to treat *Pcb* BZA12. As shown in Figure 4A, *Pcb* BZA12 cells of control had plump and complete outline. However, great deformation was observed after bacteriocin treatment (Figure 4B). Most cells exhibited a collapsed surface with a great wrinkle. Moreover, only a wizened envelope was observed for some cells. The great change of cell morphology indicates that the envelope integrity of *Pcb* BZA12 was damaged after the treatment of *L. paracasei* WX322 bacteriocin, followed by leakage of intracellular material. Damage of the cell envelope is an usual action mechanism of bacteriocins, such as Mutacin 1140 [40], AS-48 [41], and lacticin Q [42]. These bacteriocins can form pores on the cell membrane and lead to leakage of intracellular material. We infer that action mechanisms of the bacteriocin produced by *L. paracasei* WX322 to *Pcb* BZA12 is also involved in pore formation.

### 3.5. Controlling Bacterial Soft Rot of Five Kinds of Vegetables by Bacteriocin from Tomato Juice Fermentation

**Figure 5:**
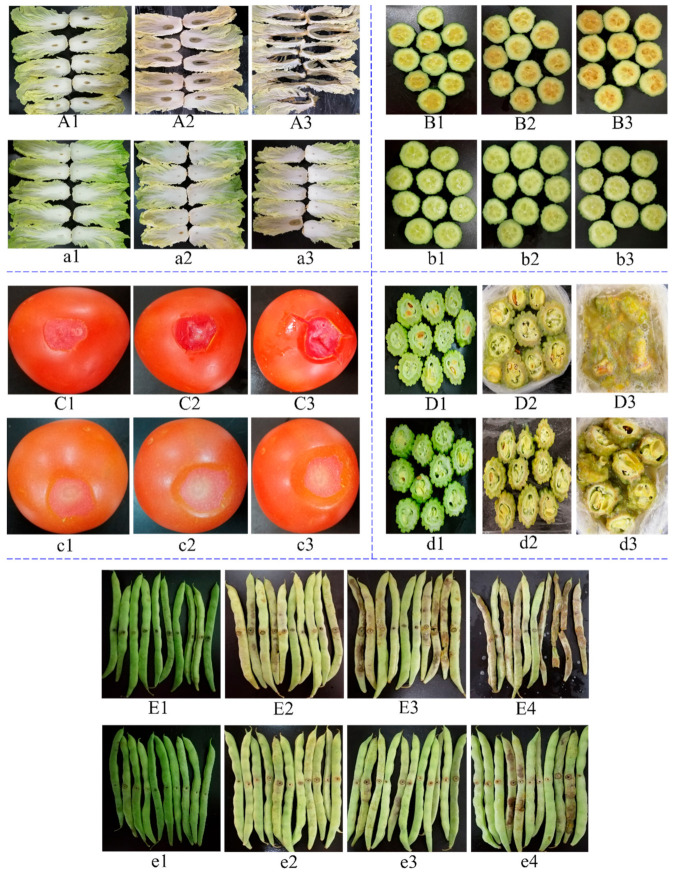
Controlling soft rot of vegetables. (**A**,**a**), Chinese cabbage; (**A1**–**A3**) are controls on 1, 2, and 3 day, respectively; (**a1**–**a3**) are treatments with bacteriocin of *L. paracasei* WX322 on 1, 2, and 3 day, respectively. (**B**,**b**), cucumber; (**B1**–**B3**) are controls on 1, 2, and 3 day, respectively; (**b1**–**b3**) are treatments with bacteriocin of *L. paracasei* WX322 on 1, 2, and 3 day, respectively. (**C**) and (**c**), tomato; C1, C2, and C3 are controls on 1, 2, and 3 day, respectively; (**c1**–**c3**) are treatments with bacteriocin of *L. paracasei* WX322 on 1, 2, and 3 day, respectively. (**D**,**d**), balsam pear; (**D1**–**D3**) are controls on 1, 2, and 3 day, respectively; (**d1**–**d3**) are treatments with bacteriocin of *L. paracasei* WX322 on 1, 2, and 3 day, respectively. (**E**,**e**), green bean; (**E1**–**E4**) are controls on 1, 2, 3, and 4 day, respectively; (**e1**–**e4**) are treatments with bacteriocin of *L. paracasei* WX322 on 1, 2, 3, and 4 day, respectively.

According to results above, bacteriocin of *L. paracasei* WX322 from tomato juice fermentation had good antibacterial activity against *Pcb* BZA12 in vitro. To further investigate its application potential as vegetable preservation in vivo, controlling effectiveness to soft rot of five vegetables were studied as shown in Figure 5. It can be found from Figure 5A,a that the bacteriocin treatment significantly inhibited the soft rot of Chinese cabbage. For the control, incidence of soft rot was 100% on the first day (Figure 5A1), then area of soft rot increased on the second day (Figure 5A2), followed by rotten leaves losing integrity and generating foul smell on the third day (Figure 5A3). However, for bacteriocin treatment, most leaves kept fresh and intact on the first day (Figure 5a1) and second day (Figure 5a2) with disease incidence of 10% ± 0% and 16.66% ± 4.71%, respectively. Incidence of soft rot was only 20% ± 8.16% on the third day (Figure 5a3). Bacterial soft rot disease, caused by *P. cartovorum*, is a fatal disease of postharvest cabbage throughout the world. Some antagonistic bacteria have been reported to control soft rot of cabbage [43], including bacteriocin-producing bacteria [44]. But the control potential of bacteriocin to soft rot of postharvest cabbage is unknown. This study can provide the evidence of biocontrol of cabbage soft rot using bacteriocin.

*P. cartovorum* is also an important pathogen causing soft rot of cucumber [45]. The soft rot pathogen, *Pcb* BZA12, used in this study was isolated from diseased cucumber [46]. Therefore, controlling soft rot of cucumber by bacteriocin was also studied. As shown in Figure 5B,b, bacteriocin from tomato juice also had a good controlling effect on soft rot of cucumber. For the control, rot and yellowing of cucumber sections occurred 100% on the first day (Figure 5B1), and symptoms aggravated on the second day (Figure 5B2) and third day (Figure 5B3). In contrast, cucumber sections kept green without rot symptom all the time during the period of 3 days (Figure 5b1–b3) after being treated by the bacteriocin sample. The result indicates that bacteriocin of *L. paracasei* WX322 from tomato juice also can be used to control soft rot of cucumber.

Soft rot disease caused by *P. carotovorum* has led to significant postharvest losses to tomatoes [47]. *P. carotovorum* is not able to penetrate the surface directly, and therefore the tomato surface was peeled off in a small area to create a wound and then the pathogen was inoculated. For the control (Figure 5C), rotten rate of tomato was 3.33% ± 4.71%, 40% ± 0%, and 70% ± 14.14% on the first, second, and third day, respectively. During which the deterioration of soft rot, symptoms of water soak and sunken tissue were increasingly serious. The peel of some tomatoes even burst (Figure 5C3). However, for bacteriocin treatment (Figure 5c), no rotten tomato was found on the first two days and disease incidence was only 13.33% ± 9.43% on the third day. The result indicates that the bacteriocin of *L. paracasei* WX322 from tomato juice performs well on controlling soft rot of postharvest tomato.

*P. carotovorum* has been reported to cause soft rot of balsam pear [48]. In this study, the result of the control (Figure 5D) demonstrated that *P. carotovorum* can cause rapid and dissolving damage to balsam pear that this vegetable rotted into pus with a terrible foul smell on the third day (Figure 5D3). Bacteriocin treatment delayed the rot to a certain degree (Figure 5d) with alleviated symptoms.

As shown in Figure 5E, *P. carotovorum* caused rot and browning to green beans with a rotten rate of 76.67% ± 4.71% for the control on the third day. On the fourth day, disease incidence reached 100% with fragmentary beans (Figure 5E4). In contrast, all green beans had intact profile and rotten rate was 26.67% ± 4.71% and 40% ± 0% on the third day (Figure 5e3) and fourth day (Figure 5e4), respectively. The result indicates that bacteriocin of *L. paracasei* WX322 from tomato juice can significantly inhibit the soft rot of green bean caused by *P. carotovorum*.

Controls of the five vegetables (Figure 5A–E) demonstrated that *P. carotovorum* can multiply in the plant tissues where it causes water soak, sinking, softening, yellowing/browning, foul smell, etc. resulting in losses of commodity value and edible value. Currently, there are few effective ways to decontaminate infected vegetables that do not pose concerns to human health [48]. However, in this study, the bacteriocin of *L. paracasei* WX322 can significantly delay the soft rot of vegetables, especially for cabbage and cucumber. Moreover, the bacteriocin was fermented in tomato juice, which is safe and healthy.

## 4. Conclusions

*P. cartovorum* is one of the most important soft rot pathogens causing great postharvest losses worldwide. Controlling soft rot is vital for the extension of vegetable shelf-life. In this study, all the 12 kinds of vegetables tested can provide nutrition for the *L. paracasei* WX322 to produce antibacterial bacteriocin, among which tomato performed best. In tomato juice, *L. paracasei* WX322 grew well with the maximum colony number around 10.19 log_10_ cfu/mL on the fifth day and the highest production of bacteriocin on the tenth day. Tomato juice can be used to replace Man-Rogosa-Sharpe broth to produce bacteriocin by *L. paracasei* WX322 because characteristics of the bacteriocin from tomato juice and from Man-Rogosa-Sharpe broth were the same. The bacteriocin killed *P. carotovorum* by causing the collapse and significant deformation of cells according to SEM images. In vivo assay, the bacteriocin of *L. paracasei* WX322 fermented in tomato juice inhibited incidence of soft rot of vegetables in different degree. This study suggests that producing bacteriocin using tomato waste (by-products and/or inferior products) can not only provide an innovative method to utilize vegetable waste, but also provide a biocontrol method to control soft rot of some postharvest vegetables.

## Figures and Tables

**Table 1:** Statistics of inhibition zone diameter of *L. paracasei* WX322 fermentation products from 12 kinds of vegetables after 5-fold concentration.

No.	Vegetables	Diameter of Inhibition Zone (mm) *
1	Tomato	47.3 ± 1.6 ^a^
2	Cucumber	42.7 ± 1.2 ^b^
3	Man-Rogosa-Sharpe broth	40.5 ± 0.4 ^c^
4	Cabbage	40.3 ± 0.4 ^cd^
5	Soybean sprout	39.9 ± 0.8 ^cde^
6	Mungbean sprout	39.4 ± 1.8 ^cde^
7	Okra	39.2 ± 0.6 ^cdef^
8	Garlic chives	38.7 ± 1.0 ^cdef^
9	Green bean	38.5 ± 1.3 ^def^
10	Carrot	38.4 ± 0.8 ^def^
11	Balsam pear	38.2 ± 0.9 ^ef^
12	White ground	38.2 ± 0.5 ^ef^
13	Purple cabbage	37.4 ± 1.2 ^e^

* Inhibition zone diameter followed by different letters are significantly different by analysis of variance (ANOVA) and Tukey’s test (*p* < 0.05).
